# Current tobacco use and COVID-19 diagnoses in a cohort of adult clients of public dental clinics in Sweden

**DOI:** 10.1038/s41598-023-28091-4

**Published:** 2023-01-21

**Authors:** M. R. Galanti, F. Andersson, I. H. Caspersen, S. Peña, S. Karvonen, P. Magnus, E. Raffetti, N. Orsini, C. Magnusson, A. N. Shaaban, M. P. Hergens, P. Skott

**Affiliations:** 1 Department of Global Public Health, Karolinska Institutet, 171 77 Stockholm, Sweden; 2 Centre for Epidemiology and Community Medicine, Stockholm Region, (CES), Solnavägen 1E (Torsplan), 113 65 Stockholm, Sweden; 3 Centre for Fertility and Health, Norwegian Institute of Public Health, Skøyen, Postbox 222, 0213 Oslo, Norway; 4 Finnish Institute for Health and Welfare, Postbox 30, 00271 Helsinki, Finland; 5 Unit for Communicable Disease Control, Postbox 6909, 102 39 Stockholm Region, Sweden; 6 Department of Orofacial Medicine, Folktandvården Stockholm, Postbox 6420, 113 82 Stockholm, Sweden

**Keywords:** Risk factors, Infectious diseases

## Abstract

Smoking has been linked with both increased and decreased risk of COVID-19, prompting the hypothesis of a protective role of nicotine in the pathogenesis of the disease. Studies of the association between use of smokeless tobacco and COVID-19 would help refining this hypothesis. We analysed data from 424,386 residents in the Stockholm Region, Sweden, with information on smoking and smokeless tobacco (*snus*) use prior to the pandemic obtained from dental records. Diagnoses of COVID-19 between February and October 2020 were obtained from health-care registers. We estimated the risk of receiving a diagnosis of COVID-19 for current smokers and for current *snus* users relative to non-users of tobacco, adjusting for potential confounders (aRR). The aRR of COVID -19 was elevated for current *snus* users (1.09 ;95%CI = 0.99–1.21 among men and 1.15; 95%CI = 1.00–1.33 among women). The risk for women consuming more than 1 can/day was twice as high as among non-users of tobacco. Current smoking was negatively associated with risk of COVID-19 (aRR = 0.68; 95% CI = 0.61–0.75); including hospital admission (aRR = 0.60; 95% CI = 0.47–0.76) and intensive care (aRR = 0.43; 95% CI = 0.21–0.89). The hypothesis of a protective effect of tobacco nicotine on COVID-19 was not supported by the findings. The negative association between smoking and COVID-19 remains unexplained.

## Introduction

The role of tobacco use in the incidence and prognosis of the coronavirus disease 2019 (COVID-19) has raised both scientific and public interest during the ongoing pandemic, due to contrasting findings reported so far in the scientific literature.

Early observations linked smoking to adverse prognosis of COVID-19 in Chinese patients^1^. Since then, studies have been published in several countries and repeatedly summarized in reviews and metanalyses, presenting a puzzling picture. On the one hand, smoking has been associated with a higher risk of adverse outcomes in COVID-19 patients admitted to hospital care. This roughly two-fold increased risk^2^ was generally in line with the previously established risk for respiratory complications caused by influenza virus^3^. However, a recent meta-analysis based on a progressive accrual of good and fair quality studies did not find an increased risk of COVID-19 among current compared with never smokers^4^. The same metanalysis also suggested that current smoking may be associated with a decreased risk of infection and/or symptomatic COVID-19^4^. In fact, earlier studies already noted substantially lower proportions of smokers among patients hospitalized for COVID-19 compared with the underlying source population, even in studies where smoking predicted a poor prognosis of the disease^5^. Some studies also reported a lower prevalence of current smoking among individuals in out-of-hospital population samples who tested positive for SARS-CoV-2 infection compared to individuals who were negative^6,7^. Several explanations have been advanced for these negative associations, detracting from a causal hypothesis of smoking being protective against infection or disease caused by SARS-CoV-2. For instance, a negative association may appear due to selection bias, based on different characteristics (e.g., occupation) of individuals being tested and/or hospitalized. Information bias, due to under-ascertainment or under-report of smoking among hospitalized patients is another possibility^8^.

However, a causal link between smoking and a decreased risk of infection with SARS-CoV-2 and/or symptomatic COVID-19 has also been postulated, implying a protective role of nicotine in the pathogenesis of the disease. First, nicotine upregulates the angiotensin-converting enzyme 2 (ACE2) receptors in the lung, thus potentially increasing the virus entry points^9^. However, ACE2 receptors are also involved in the homeostatic regulation of the renin-angiotensin system (RAS), reducing the risk for pulmonary oedema and inflammation^10^. Recent studies even suggested that ACE2 can be suppressed by exposure to smoking^11^, for instance by activation of aryl-hydrocarbon receptor (AHR) due to polycyclic aromatic hydrocarbons^12^. Second, nicotine binding to the nicotine acetylcholine receptors (nAchR) in the lungs may downregulate the inflammatory response underlying the dramatic respiratory impairment typical of the disease (cytokine storm)^13^. It has also been proposed that nitric oxide (NO), a gas contained in the inhaled smoke, might have a toxic effect on the virus^14^. The hypothesized mechanisms remain speculative and lack empirical support in humans.

Given the public health importance of tobacco use as a risk factor for morbidity and mortality, but also of the potential therapeutic role of medicinal nicotine, it is very important that any claim of causality would rest on large population studies with low risk for bias, as also endorsed by the WHO^15^. One way to refine this hypothesis would be to analyze the association between smokeless tobacco use and COVID-19.

In Sweden, the use of the oral moist snuff known as *snus* is common^16^. This tobacco type doesn’t impact on the respiratory system but contains nicotine in the same concentration per gram of tobacco as cigarettes, albeit with a different profile of absorption^17^. Along with the lower level of restriction of use this makes the *snus* user potentially exposed to sustained high levels of nicotine. Therefore, we explored the association between tobacco use (cigarette smoking and the Swedish smokeless tobacco *snus*) and COVID-19 in a longitudinal study based on a historical cohort of clients of the public dental clinics in the region of Stockholm, Sweden.

Based on the a priori knowledge of the hazards connected to tobacco use, we hypothesized that:

the risk of COVID-19 and of severe cases requiring intensive care or resulting in death would be higher among current smokers than among current non-smokers; the risk of COVID-19 would not differ between current *snus* users and current non-users.

## Methods

The study protocol of this retrospective cohort study was pre-registered at clinicaltrials.gov (NCT04896918, ID 4-1457/2021).

All methods were carried out in accordance with relevant guidelines and regulations.

The Ethical Review Authority of Sweden approved the study protocol, decision 2020-07152. In this decision, the requirement of individual informed consent was waived in accordance with the current practice of register-based research in Sweden handling unidentified personal information.

### Study population and analytical sample

We identified a historical cohort of clients of public dental clinics in the Stockholm region. In Sweden, the public dental clinics (Folktandvården, FTV) routinely provide preventive visits (oral check-ups) to all residents who choose to receive care in these clinics. It is estimated that in the Stockholm region about 30% of the adult population of about 1.9 million inhabitants 18 years or older is enlisted in the 79 public clinics (the remaining being clients of private clinics). At each health check-up, self-reported information is collected on lifestyle and co-morbidities with relevance for oral health, with a uniform instrument in use since October 2015 (health declaration). Smoking and *snus* use is ascertained as past use, current use, and amount of current use. In late February 2020, the usual routine activity of the clinics was disrupted by the pandemic, and the oral health check-up was discontinued. We initially identified records corresponding to unique adult individuals accessing public dental clinics in the Stockholm Region between October 2015 to January 2020. Clients of these clinics are considered adults from the age of 23 years. Individuals accessing the clinics within seven months from the onset of the pandemic in Sweden (1st July 2019–31st January 2020), were considered to represent recent tobacco use experience, not likely to have changed before the pandemic. We used the national personal numbers assigned to every resident in Sweden at birth or at immigration to obtain information on diagnoses of COVID-19 and tobacco-related diseases among individuals in this cohort through record-linkage with the regional database of inpatient and outpatient health care (VAL database). Demographic information was extracted through record-linkage with the register of the total population of the region of Stockholm held by Statistics Sweden.

**Figure 1:**
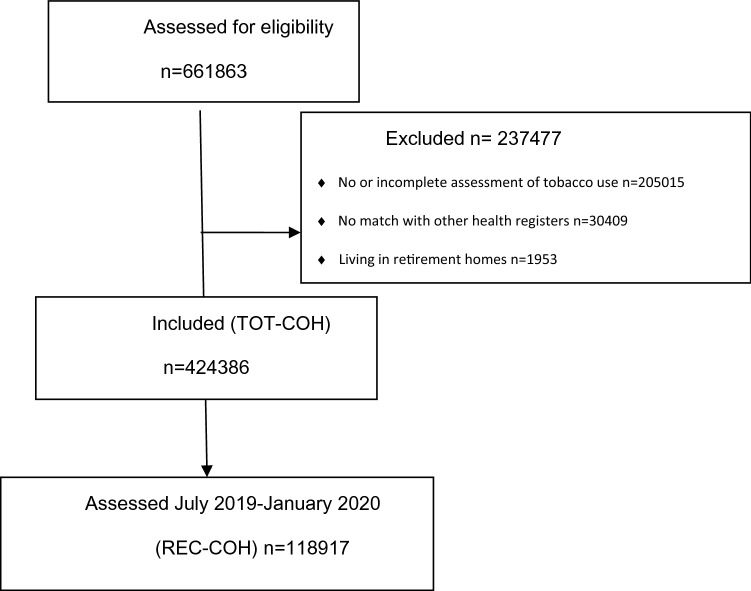
Flow diagram of participants.

Only individuals with complete information on both smoking and *snus* use who could be linked to the regional registers were included in the analysis. Residents in assisted elderly dwellings were excluded because these individuals represent a segment of the population affected by multiple disabilities, very high mortality rates for all causes, and probably very low prevalence of tobacco use. After these exclusions, there were 424,386 individuals in the whole cohort (TOT-COH), of which 118,917 represented recent users (REC-COH). Figure 1 displays the flowchart of the analytical samples.

### Information

#### Exposure variables (current tobacco use)

Current tobacco use was reported in the dental clinic checklist separately for cigarette smoking and for *snus* use. It was analyzed both as a dichotomous variable (yes/no) and as a categorical variable of the originally reported average daily consumption *(*for cigarettes: no smoking, 10 cigarettes per day or less (CPD); 11–20 CPD; more than 20 CPD; for *snus* use: no use, less than 1/2 can per day; ½–1 can per day; more than 1 can per day).

Tobacco use was categorized as no current use; current exclusive smoking; current exclusive *snus* use; current mixed use (smoking and *snus*).

In a quality assessment, the variable corresponding to past tobacco use was found very incomplete because of inconsistent assessment; therefore it was not included in the analysis. This implies that former users of tobacco are included in the category of “current non-users”.

#### Outcome variables (diagnosis of COVID-19)

Information on the outcome was accrued for the period of February 25 -October 22, 2020.

Four outcome variables are included in the analysis:

- Any diagnosis of COVID-19, whether in hospital or outside*,* consisting of at least a positive polymerase chain reaction test (PCR) reported by the laboratories to Sweden´s national electronic surveillance system for communicable diseases, SmiNet.
- Hospital admission with a diagnosis of COVID-19 (ICD-10 codes U071 and U072). The diagnosis could be registered either as a main or as a concomitant diagnosis.
- Admission to an intensive care unit (ICU) because of a diagnosis of COVID-19 (ICD-10 codes as above).
- Death by COVID-19, established using the Swedish Cause of Death Registry, which is based on the death certificate filled in by physicians. All deaths occurring during the follow-up period with COVID-19 registered as the main cause were included*.* The restriction to main cause was done to maximize the specificity of the diagnosis^18^.

The outcome variables described above are not mutually exclusive, i.e., a includes b, c and d; b includes c and d; c includes at least some d; therefore any step can be conceptualized as a case of progression from infection with SARS-CoV-2 virus (see also Supplementary material Fig. 2).

#### Other covariates

Other variables included in the analysis as potential confounders or as moderators were:

- Socio-demographic: gender; age; country of birth (Sweden; other Nordic country; other country); education (compulsory school, i.e., nine years of schooling; high school, i.e. 2 or 3 years of schooling after the compulsory education; university); occupation categorized as “high”, “moderate” or “low” risk of SARS-CoV-2 infection based on “a priori” knowledge on exposure to transmission of the virus; household disposable income in Swedish crowns—SEK per year; cohabitation with others (yes/no). We considered age, education, occupation, income, and cohabitation at the start of follow-up.
- Chronic diseases that have been causally associated with tobacco use: any diagnosis of cancer, COPD, cardiovascular disease, diabetes mellitus, and Parkinson's disease up to February 25, 2020.

### Statistical analysis

Risk ratios (RR) and their corresponding 95% CI were estimated through generalized linear models (GLM) for the binomial family with log link and the maximum likelihood optimization algorithm. The models were estimated for the following events: any diagnosis of COVID-19 (positive PCR test); hospitalization; admission to intensive care; and death because of COVID-19. These events were considered both in a cumulative fashion (i.e., “any diagnosis” includes even hospitalized, intensive care, and deaths) and as mutually exclusive events, i.e., extra-hospital diagnoses with non-lethal outcome; hospital diagnoses with no intensive care; intensive care surviving the disease; deaths. In the primary analyses, we separately compared current exclusive smokers or current exclusive users of *snus* with current non-users of any tobacco (reference). In a secondary analysis (sensitivity analysis), we also estimated the risk of COVID-19 among mixed tobacco users (those currently using both cigarettes and snus) compared with current non-users.

It should be noted that among these latter, some individuals may have been former tobacco users.

Because of missing information, the analysis of dose–response with reported tobacco consumption was based on slightly lower numbers of individuals.

Adjustments were made for putative confounders, namely age (in continuous form), gender, country of birth, type of employment, education, income (continuous), and cohabitation. The a priori consideration of the confounding structure is shown as directed acyclic graphs (DAGs) in Fig. S1 (Supplementary material), where different assumptions were made concerning the role of chronic diseases that have been causally associated with tobacco use (parts a and b). To explore possible effect modification due to different patterns of exposure and/or outcomes as well as for sensitivity analyses we conducted pre-registered separate analyses by age group (below 70 years of age or 70 years and above, cutoff chosen because of the sharp increase in mortality from COVID-19 after the age of 70; sex (in analyses of *snus* use); presence of chronic diseases causally associated with tobacco use; assumed infection risk associated with occupation (high vs. moderate and low); recency of assessment of tobacco use (more than 7 months or 7 months or less before the onset of the pandemic); and pandemic period (up to May 31, 2020; from June 1 until end of follow-up). This latter categorization corresponded to different strategies of virologic testing in Sweden, very restricted (only on medical prescription) in the first period, available on individual request thereafter. In a further sensitivity analysis, we used the national and regional incidence of PCR tests and the proportion of positive tests in the general population to estimate the expected number of smokers and non-smokers with a positive PCR test in the study cohort, under the null hypothesis of no difference in the risk of COVID-19. The expected and observed numbers were then compared to explore the extent of a potential under-ascertainment of COVID-19 among smokers. All analyses were performed with Stata version 16.1.

## Results

Baseline characteristics of cohort participants are shown in Table 1, separately for the whole sample (TOT-COH) and for the sample of individuals whose tobacco use was assessed during the 7 months immediately preceding the first case of COVID-19 (REC-COH).

**Table 1:** Socio-demographic characteristics, tobacco use and incident diagnoses of COVID-19 February 25–October 22, 2020, among clients of the public dental care in Stockholm Region.

	TOT-COH^a^ N = 424,386 (%)	REC-COH^a^ N = 118,917 (%)
Sex		
Male	192,224 (45.3)	53,330 (44.9)
Female	232,162 (54.7)	65,587 (55.2)
Age mean (SD)	46.5 (15.8)	47.7 (16.7)
Education		
Compulsory (9 years)	34,581 (8.2)	9470 (8.0)
High school (12 years)	142,866 (33.7)	40,541 (34.1)
University (> 12 years)	237,422 (55.9)	65,474 (55.1)
Missing	9517 (2.1)	3432 (2.8)
Occupational risk for infection		
Low	256,169 (60.4)	72,326 (60.8)
Moderate	76,157 (17.9)	20,831 (17.5)
High	56,173 (13.2)	15,491 (13.0)
Missing	35,887 (8.5)	10,269 (8.7)
Disposable yearly income in Swedish crowns, mean (SD)	269,869.4 (408,014.9)	270,076.2 (275,608.2)
Cohabitation		
Yes	329,668 (77.7)	91,153 (76.7)
No	89,399 (21.1)	26,489 (22.3)
Missing	5319 (1.2)	1275 (1.0)
Country of birth		
Sweden	335,900 (79.2)	95,528 (80.3)
Other Nordic countries	11,838 (2.8)	3558 (3.0)
Other countries	68,863 (16.2)	17,868 (15.0)
Missing	7785 (1.8)	1963 (1.7)
Current smoking		
No	386,746 (91.1)	109,191 (91.8)
Yes (all)	37,640 (8.9)	9726 (8.2)
≤ 10 cig/day	24,071 (5.7)	6181 (5.2)
11–20 cig/day	10,459 (2.5)	2498 (2.1)
> 20 cig/day	1455 (0.3)	349 (0.3)
Missing	2378 (0.6)	919 (0.8)
Current use of *snus*		
No	373,328 (88.0)	105,289 (88.5)
Yes (all)	48,809 (11.5)	13,481 (11.3)
< 1/2 can/day	28,365 (6.7)	7932 (6.7)
½–1 cans/day	17,919 (4.2)	4777 (4.0)
> 1 cans/day	2039 (0.5)	507 (0.4)
Missing	3640 (0.9)	699 (0.6)
Previous diagnoses of tobacco-related diseases	114,592 (27.0)	35,532 (29.9)
Infection of SARS-CoV-2/COVID-19	6540 (1.5)	1820 (1.5)
Hospital admissions with COVID-19	1411 (0.3)	415 (0.4)
Intensive care for COVID-19	184 (0.04)	51 (0.04)
Death with a diagnosis of COVID-19	205 (0.05)	64 (0.05)

^a^TOT-COH = Whole sample REC-COH = recent cohort, i.e., individuals accessing the dental clinics 7 months or less from the onset of the pandemic in Sweden (1st July 2019–31st January 2020).

Compared with the whole regional population, clients in public dental care were on average 3 years younger, with a higher proportion of women, individuals with university education, and Swedish born (Supplementary material Table S1). The proportion of current smokers was around 9% and that of *snus* users about 11%, in line with the proportions of daily smokers/*snus* users in previous regional or national surveys^19^. Most smokers smoked 10 cigarettes per day or less, with very few individuals smoking more than a pack per day. Among *snus* users, about 66% consumed less than half a can per day. There were no appreciable differences between the whole cohort of clients and the most recent clients (Table 1).

The cumulative incidence of COVID-19 diagnoses (1.5%) and of death (0.05%) were similar to the underlying regional population in the same period (1.2% and 0.09%, respectively), considering the age and sex differences.

Table 2 reports the distribution of incident COVID-19 diagnoses, hospital admissions, admission to ICU, and deaths across categories of current tobacco use. There were very few events in the category of mixed users, especially concerning hospital admission, intensive care, and deaths.

**Table 2:** COVID-19 incident outcomes February 25–October 22, 2020 among clients of the public dental care in Stockholm Region, by categories of tobacco use.

Current tobacco use	Infection with SARS-CoV-2/COVID-19	Hospital admission with COVID-19	Hospital intensive care for COVID-19	Death by COVID-19
	N (column %)	N (column %)	N (column %)	N (column %)
No use	5383 (82.3)	1216 (86.1)	155 (84.2)	181 (88.3)
Exclusive cigarette smoking	372 (5.7)	74 (5.4)	9 (4.9)	14 (6.8)
Exclusive use of *snus*	694 (10.6)	102 (7.2)	16 (8.7)	7 (3.4)
Mixed use (cigarettes and *snus*)	58 (0.9)	9 (0.6)	3 (1.6)	2 (1.0)
Missing	33 (0.5)	10 (0.7)	1 (0.5)	1 (0.5)
Total cases	6 540	1 411	184	205

The risk of being diagnosed with COVID-19 throughout the study period was lower for current smokers relative to current non-users of tobacco, both before (not shown) and after adjustment for potential confounders (Table 3). Of note, the results were unchanged when the presence of chronic diseases (27% of the sample) was adjusted for (not shown). In addition, there was a hint of dose–response with decreasing risk associated with reported increasing consumption of cigarettes per day.

**Table 3:** Adjusted^a^ Risk Ratio (RR) and 95% Confidence Intervals (CI) of COVID-19 for current exclusive smokers compared to non-users of tobacco among clients of the public dental care clinics in Stockholm region (n = 365,627).

Current tobacco use	Diagnoses of COVID-19 (all)	Hospital admission	Intensive care	Death
	RR (CI)	RR (CI)	RR (CI)	RR (CI)
	N = 5595	N = 1244	N = 158	N = 189
Non-user of tobacco	(ref)	(ref)	(ref)	(ref)
Current smoker (all)	0.68 (0.61–0.75)	0.60 (0.47–0.76)	0.43 (0.21–0.89)	1.04 (0.59–1.84)
≤ 10 cig./day	0.80 (0.71–0.90)	0.63 (0.46–0.85)	0.58 (0.26–1.33)	0.75 (0.31–1.83)
11–20 cig./da	0.44 (0.35–0.56)	0.52 (0.33–0.80)	0.16 (0.02–1.16)	1.24 (0.51–3.03)
> 20 cig./day	0.28 (0.13–0.63)	0.29 (0.07–1.16)	NE	NE

*NE* not estimated because of the low number of events.

^a^Adjusted for sex, age (continuous), education, income (continuous), occupational risk, country of birth, and cohabitation.

The associations were in the same direction for the incidence of hospital admission and admission to hospital intensive care, albeit in this latter case, the precision was low. The estimated relative risk of death for current smokers compared to non-user of tobacco was compatible with the hypothesis of no effect (RR 1.04, 95% CI 0.59–1.84).

In Table 4, the relative risk of COVID-19 diagnoses and death is shown for exclusive users of *snus* compared to non-users of tobacco, separately for women and men. Among men, there was an estimated 9% higher (95% CI = 0.99–1.21) risk of COVID-19 for current *snus* users vs non-users of tobacco. Among women *snus* users, there was a 15% higher risk (95% CI = 1.00–1.33), indeed two-fold higher among the minority consuming more than 1 can per day (adjusted RR 2.21, 95% CI = 1.17 -4.20). The associations with hospital admission, intensive care, or death were very imprecise in both sexes, and no further patterns were apparent.

**Table 4:** Adjusted^a^ Risk ratio (RR) and 95% Confidence Intervals (CI) of COVID-19 for current exclusive *snus* users compared to non-users of any tobacco among clients of the public dental clinics in Stockholm region (TOT-COH, N_men_ = 169,488, N_women_ = 205,831).

Current tobacco use	Diagnoses of COVID-19 (all)	Hospital admission	Intensive care	Deaths
	RR (CI)	RR (CI)	RR (CI)	RR (CI)
Men	N = 2459	N = 642	N = 113	N = 116
No tobacco use	(ref)	(ref)	(ref)	(ref)
Current use of *snus* (all)	1.09 (0.99–1.21)	0.95 (0.75–1.21)	0.85 (0.47–1.53)	0.64 (0.28–1.49)
< 1/2 can/day	1.05 (0.92–1.19)	0.91 (0.66–1.24)	0.82 (0.38–1.79)	0.69 (0.25–1.90)
½–1 can/day	1.11 (0.96–1.28)	0.97 (0.67–1.40)	0.84 (0.34–2.10)	0.63 (0.15–2.61)
> 1 can/day	1.22 (0.84–1.78)	1.02 (0.38–2.74)	1.36 (0.19–9.85)	NE

Women	N = 3460	N = 629	N = 53	N = 66
No tobacco use	(ref)	(ref)	(ref)	(ref)
Current use of *snus* (all)	1.15 (1.00–1.33)	1.13 (0.72–1.77)	1.62 (0.50–5.29)	NE
< 1/2 can/day	1.13 (0.95–1.34)	1.15 (0.68–1.97)	1.59 (0.38–6.63)	NE
½–1 can/day	1.18 (0.91–1.53)	1.18 (0.53–2.64)	1.85 (0.25–13.57)	NE
> 1 can/day	2.21 (1.17–4.20)	NE	NE	NE

*NE* not estimated because of the low number of events.

^a^Adjusted for age (continuous), education, income (continuous), occupational risk, country of birth, and cohabitation.

The risk ratios for current mixed users of cigarettes and *snus* were similar to those for smokers (Supplementary Table S7).

When the outcome was analyzed in mutually exclusive categories of COVID-19 outcomes, similar patterns of associations were found. Among current smokers compared with non-users of tobacco, the adjusted risk ratio of a diagnosis of COVID-19 (positive PCR) that did not require hospital admission and did not cause death was 0.72 (95% CI 0.64–0.81); the corresponding aRR for hospital admission not requiring intensive care and not exiting in a death was 0.58 (95% CI 0.44–0.76); and for intensive care that was not followed by death was 0.46 (0.21–1.00).

### Subgroup and sensitivity analyses

The results were virtually unchanged in the analysis of the cohort with more recent assessment of tobacco use (REC-COH) (Supplementary material Table S2).

The negative association reported in Table 3 was only observed among individuals younger than 70 years (Supplementary material Table S3).

The association between smoking and COVID-19 (all diagnoses and hospital admissions) was in the same direction as described in Table 3 in both time periods (up to May 31 or from 1 June 2020 on), although somewhat stronger during the early phase of the pandemic (Supplementary material Table S4).

The associations in Table 3 were virtually the same among individuals whose occupation entailed high or low-moderate risk of infection, respectively (Supplementary material Table S5).

Smoking was equally associated with a lower risk of any diagnosis and of hospital diagnosis with COVID-19 among individuals with or without previous diagnoses of chronic diseases causally associated with tobacco use (Supplementary material Table S6). In particular, the relative risk of hospital admission with COVID-19 was very low for smokers compared with non-users of tobacco among individuals without chronic diseases.

The number of smokers in the study cohort that was expected to be infected by SARS-CoV-2 or to develop COVID-19 during the study period, if they were tested with the same frequency as the general population, under the null hypothesis of no difference in risk was estimated as 414 (observed number 434, i.e., 4.4% higher). The same applied to non-smokers (expected 4257, observed 6116, i.e., 30.4% higher). Therefore, we estimated that the difference in the incidence of tests between smokers and non-smokers should have been larger than 25% to generate the observed risk ratios assuming no difference in true risk of COVID-19 between smokers and non-smokers, and even larger under the hypothesis of a higher risk among smokers.

## Discussion

In this large cohort accrued from clients of public dental services before the onset of the COVID-19 pandemic current smoking was associated with a decreased likelihood of being diagnosed with COVID-19, after adjustment for several potential confounders. *Snus* use, on the other hand, was associated with a higher risk of COVID-19 particularly among women heavy users. The risks of hospital admission and of intensive care because of COVID-19 followed the same patterns, while the risk of death was not associated with tobacco use in this cohort. The negative association between smoking and COVID-19 has been described. A very comprehensive meta-analysis that progressively summarizes new studies^4^ presented inference in line with that obtained in this study. To the best of our knowledge, an analysis of the Swedish smokeless tobacco (*snus*) use in relation to COVID-19 was previously reported only in an article from Finland^20^, with similar results. *Snus* is a tobacco product that is completely lacking an impact on the respiratory system but delivers substantial doses of nicotine to the user^17^. Therefore, the results of this study do not accord with the hypothesis of a beneficial role of nicotine in infection or disease caused by the SARS-CoV-2 virus. Nevertheless, the hypothesis of a role of nicotine in the risk of COVID-19 cannot be completely dismissed solely based on the positive association between *snus* use and COVID-19 presented in this study. The confounding structure of the association between *snus* and COVID-19 may be different from that hypothesized for smoking, and it is a possible explanation for the diverging associations. Another possibility is that the risk of infection and/or the prognosis of the disease among tobacco users could be mediated by characteristics or lifestyle that we could not explore in this study. For instance, high body mass index (BMI) has been implicated in the prognosis of COVID-19^21^, and BMI is on average lower among smokers than among non-smokers^22^ and higher among *snus* users than among non-users^23^. Also, the manipulation of the product requires frequent finger-mouth cavity contacts among *snus* users, which may expose them to the risk of infection. Unlike smoking, *snus* use is not forbidden indoors, and *snus* users are not perceived as individuals at risk of severe consequences of COVID-19. Therefore, they may be less discouraged than smokers from attending places where the risk of transmission is high (e.g., workplaces, and parties). It should be noted that during the pandemic period included in this analysis there were no stringent recommendations in Sweden concerning the use of face masks. Finally, the frequency of testing during the pandemic may be higher among *snus* users than in the general population, as was suggested in a parallel study from Norway (work submitted)^24^. All these factors may theoretically obscure a potential negative association between *snus* and COVID-19 due to nicotine. However, findings speaking against a protective role of oral tobacco or nicotine were also presented in other recent studies. In a cross-sectional study conducted in Indiana (USA), the prevalence of past or current infection with SARS-CoV-2 was higher among users than among non-users of chewed tobacco^25^. Nicotine did not show cytoprotective effects against SARS-CoV-2 in vitro assays^26^. In a clinical trial the use of medicinal nicotine as adjuvant treatment in COVID-19 patients admitted to intensive care units did not affect mortality^27^.

The conundrum of a possible causal negative association between current smoking and COVID-19 remains unanswered. Against this possibility speaks the counter-intuitive pathway linking a major hazard for the respiratory system to the decreased occurrence of a disease that has its major clinical expression in the same system^28^. The impact of a bias linked to differential referral to health care (e.g., virology test) among smokers compared with non-smokers cannot be excluded because in this cohort we did not have information on the incidence of testing. In previous studies, both higher^4^, lower^29^ or no different^30^ testing behavior among smokers compared with non-smokers have been described. In a related article based on a Norwegian cohort, there was no difference in self-reported testing behavior between smokers and non-smokers (work submitted)^24^.

Of note, in this population-based study, a lower (but not a higher) test opportunity among smokers compared with non-smokers (assuming no true risk difference) could result in a non-causal negative association between smoking and COVID-19, due to a relative under-ascertainment of COVID-19. Sensitivity analyses suggested indeed that the incidence of testing in this cohort may have been somewhat lower among smokers than among non-smokers. However, the magnitude of the under-ascertainment should be substantial to result in the negative association found in this data, especially in the case of a true higher incidence of the disease among smokers.

Also, the similarity of the results in two different periods of the pandemic, where testing strategies were completely different; and the similarity of the associations across stages of severity of the disease speak against a substantial bias introduced by testing opportunities.

Another possibility for a non-causal negative association between smoking and COVID-19 diagnoses may be a relatively lower sensitivity of the test of choice (SARS-CoV-2 RNA polymerase chain reaction) among current smokers^31^.

Finally, smokers may be protected from severe manifestations of the disease caused by SARS-CoV-2 because of their frequent exposure to other respiratory viruses, including other Coronaviruses, with consequent development of cross-immunity^32^.

Additional limitations should be kept in mind when interpreting the results from this study. We cannot rule out selection bias (for instance, if tobacco users and non-users in this cohort would be different from their counterparts in the source population because of factors differentially predicting the outcome). We could not explore whether specific mediators may account for the different association seen among smokers and *snus* users. Further, the analysis of former smokers (who have been found at higher risk for COVID-19)^4^ was not possible in this cohort. The lack of information on former smokers forced their inclusion in the reference category of “non-current tobacco use”, which may have contributed to a spurious lower risk observed for current smokers.

Notwithstanding these limitations the study has several strengths. First, it relies on a pre-registered analysis protocol, being part of an international consortium in the Nordic Countries^20,24^ triangulating the same scientific question^33^. Second, the large study cohort was population based, thus avoiding selection related to the ascertainment of the disease. Third, smoking and *snus* use were prospectively assessed with respect both to the disease outcome and to the onset of the pandemic, during which behavioral changes may have occurred. The exposure also included quantitative information on habitual consumption that allowed the study of dose–response. Fourth, the outcome was assessed through the regional surveillance system for COVID-19 and through health care registers, that have been checked for quality and uniformity of reports. In addition, COVID-19 outcomes could be studied according to indicators of disease progression and course, i.e., from out-of-hospital infection to death. Finally, we had the possibility to account for several confounders, among which the occupational and social risk of infection, rendering residual confounding less likely than in previous studies. A recent study from the UK employing both a traditional epidemiologic approach and a mendelian randomization analysis suggested indeed residual confounding as a likely explanation for the negative association smoking-COVID-19^34^.

We conclude that the purported causal protective effect of tobacco nicotine is not supported by the present study. The counter-intuitive lower risk of infection and of severe COVID-19 among smokers is still to be explained.

## Supplementary Information

Supplementary Information.

## Data Availability

Individual information in this study is not publicly available but can be accessed with a research proposal and ethical clearance from the Swedish Ethical Review Authority. Meta-data and codebooks can be made available upon request to the corresponding author.
