# Supplementary material for: Current tobacco use and COVID-19 diagnoses in a cohort of adult clients of public dental clinics in Sweden
Source: Sci Rep. 2023 Jan 21;13:1204. doi: 10.1038/s41598-023-28091-4 (PMC9862224; doi:10.1038/s41598-023-28091-4)

**SUPPLEMENTARY MATERIAL**

**Galanti MR et al. Current tobacco use and COVID-19 diagnoses in a cohort of adult clients of public dental clinics in Sweden**

**Correspondence:**

Maria Rosaria Galanti

Karolinska Institutet

Solnavägen 1E (Torsplan), 113 65 Stockholm,

SWEDEN

Cell phone: +46 70 4718984

Email: rosaria.galanti@ki.se

[Table S1. Comparison of selected demographic characteristics between the study cohort and the general adult population in the region of Stockholm 3](#_Toc87141194)

[Table S2. Adjusted risk ratio (RR) of COVID-19 for smokers and snus users compared to non-users of tobacco among cohort participants whose tobacco use was assessed in the 7 months closest to the pandemic (REC-COH, Nsmoke = 102 351, Nsnus,men = 47 648, Nsnus,women = 58 401) 4](#_Toc87141195)

[Table S3. Adjusted risk ratio (RR) of COVID-19 for smokers compared to non-users of tobacco, by age groups (N_23-69_ = 323 413, N_70+_ = 41 951) 5](#_Toc87141196)

[Table S4. Adjusted risk ratio of COVID-19 for smokers compared to non-users of tobacco, by time period^3^ during the pandemic (N_01/02-31/05_ = 365 600, N_01/06-30/10_ = 365 496) 6](#_Toc87141197)

[Table S5. Adjusted risk ratio of COVID-19 for smokers compared to non-users of tobacco, by occupational risk of infection (N_low and middle_ = 291 479, N_high_ = 49 878) 7](#_Toc87141198)

[Table S6. Adjusted risk ratio of COVID-19 for smokers compared to non-users of tobacco among individuals with or without selected diagnoses^2^ of tobacco-related diseases (N_no diagnosis_ = 264 005, N_diagnosis_ = 101 622) 8](#_Toc87141199)

[Table S7. Adjusted Risk Ratio (RR) and 95% Confidence Intervals (CI) of COVID-19 for current smokers, users of snus and mixed users compared to non-users of tobacco among clients of the public dental care clinics in Stockholm region (N = 411 111) 9](#_Toc87141200)

[Figure S1. DAG of the hypothesized causal link between tobacco use and COVID-19 10](#_Toc87141201)

# Table S1. Comparison of selected demographic characteristics between the study cohort and the general adult population in the region of Stockholm

|  | **Study cohort**  **N= 424 386** | **Region of Stockholm**  **N = 1 720 330** |
| --- | --- | --- |
| **Sex** |  |  |
| Male | 192 224 (45.3%) | 854 426 (49.7%) |
| Female | 232 162 (54.7%) | 865 904 (50.3%) |
| **Age** mean (SD) | 46.5 (15.8) | 49.1 (17.1) |
| **Education** |  |  |
| Compulsory (9 years) | 34 581 (8.2%) | 200 755 (11.7%) |
| High school (12 years) | 142 866 (33.7%) | 604 063 (35.1%) |
| University (>12 years) | 237 422 (55.9%) | 796 030 (46.3%) |
| Missing | 9 517 (2.1%) | 119 482 (6.9%) |
| **Occupational risk for infection** |  |  |
| Low | 256 169 (60.4%) | 1 002 284 (58.3%) |
| Moderate | 76 157 (17.9%) | 300 752 (17.5%) |
| High | 56 173 (13.2%) | 200 046 (11.6%) |
| Missing | 35 887 (8.5%) | 217 248 (12.6%) |
| **Disposable yearly income in Swedish crowns, mean (SD)** | 269 869.4 (408 014.9) | 268 622.7 (834 349.4) |
| **Cohabitation** |  |  |
| Yes | 329 668 (77.7%) | 1 293 166 (75.2%) |
| No | 89 399 (21.1%) | 377 387 (21.9%) |
| Missing | 5 319 (1.2%) | 49 777 (2.9%) |
| **Country of birth** |  |  |
| Sweden | 335 900 (79.2%) | 1 173 975 (68.3%) |
| Other Nordic Countries | 11 838 (2.8%) | 53 912 (3.1%) |
| Other countries | 68 863 (16.2%) | 424 753 (24.7%) |
| Missing | 7 785 (1.8%) | 67 690 (3.9%) |

# Table S2. Adjusted^1^ risk ratio (RR) of COVID-19 for smokers and snus users compared with non-users of tobacco among cohort participants whose tobacco use was assessed in the 7 months closest to the pandemic (REC-COH, Nsmoke = 102 351, Nsnus,men = 47 648, Nsnus,women = 58 401)

| Cigarette smoking | All diagnoses of Covid-19 | Hospital admission | ICU admission | Death |
| --- | --- | --- | --- | --- |
|  | RR (CI) | RR (CI) | RR (CI) | RR (CI) |
|  | N= 1554 | N= 374 | N= 42 | N= 62 |
| No tobacco use | (reference) | (reference) | (reference) | (reference) |
| Smoking (all) | 0.64 (0.51-0.79) | 0.58 (0.36-0.92) | 0.45 (0.11-1.89) | 1.75 (0.74-4.14) |
| <=10 cig./day | 0.77 (0.61-0.99) | 0.64 (0.36-1.15) | 0.41 (0.06-2.97) | 1.67 (0.52-5.40) |
| 11-20 cig./day | 0.34 (0.20-0.58) | 0.29 (0.09-0.91) | NA | 0.93 (0.13-6.77) |
| >20 cig./day | NE | NE | NE | NE |
| Snus use | All diagnoses of Covid-19 | Hospital admission | ICU admission | Death |
|  | RR (CI) | RR (CI) | RR (CI) | RR (CI) |
| **Men** | N= 676 | N= 178 | N= 27 | N= 33 |
| No tobacco use | (reference) | (reference) | (reference) | (reference) |
| Snus (all) | 1.02 (0.84-1.24) | 0.84 (0.51-1.39) | 1.22 (0.41-3.64) | 0.34 (0.05-2.57) |
| <=1/2 can/day | 0.93 (0.71-1.20) | 0.57 (0.27-1.22) | 1.60 (0.47-5.46) | 0.56 (0.08-4.14) |
| 1/2-1 can/day | 1.21 (0.93-1.59) | 1.41 (0.76-2.63) | 0.81 (0.11-6.16) | NA |
| >1 can/day | 0.74 (0.28-1.97) | NE | NE | NE |
| **Women** | N= 975 | N= 202 | N= 20 | N= 24 |
| No tobacco use | (reference) | (reference) | (reference) | (reference) |
| Snus (all) | 1.22 (0.94-1.59) | 1.25 (0.58-2.67) | 4.01 (1.12-14.39) | NE |
| <=1/2 can/day | 1.29 (0.95-1.75) | 1.03 (0.38-2.78) | 3.84 (0.85-17.29) | NE |
| 1/2-1 can/day | 1.24 (0.77-2.00) | 1.99 (0.63-6.26) | 5.03 (0.65-39.19) | NE |
| >1 can/day | 2.29 (0.75-7.00) | NE | NE | NE |
|  |  |  |  |  |

^1^Adjusted for sex, age (continuous), education, income (continuous), occupational risk, country of birth and cohabitation

NE= Not estimated because of low numbers of events

# Table S3. Adjusted^1^ risk ratio (RR) of COVID-19 for smokers compared with non-users of tobacco, by age groups (N_23-69_ = 323 413, N_70+_ = 41 951)

| **Age group**  Tobacco use | All diagnoses of Covid-19 | Hospital admission | ICU admission | Death |
| --- | --- | --- | --- | --- |
|  | RR (CI) | RR (CI) | RR (CI) | RR (CI) |
| **23-69 years** | N= 5 036 | N= 808 | N= 131 | N= 47 |
| No tobacco use | (reference) | (reference) | (reference) | (reference) |
| Smoking (all) | 0.68 (0.61-0.76) | 0.51 (0.38-0.68) | 0.38 (0.18-0.82) | 0.40 (0.12-1.31) |
| <=10 cig./day | 0.81 (0.72-0.92) | 0.57 (0.40-0.81) | 0.61 (0.27-1.39) | 0.53 (0.13-2.20) |
| 11-20 cig./day | 0.42 (0.32-0.54) | 0.40 (0.23-0.69) | NE | 0.37 (0.05-2.71) |
| >20 cig./day | 0.26 (0.11-0.62) | 0.15 (0.02-1.10) | NE | NE |
| **70+ years** | N= 559 | N= 436 | N= 27 | N= 142 |
| No tobacco use | (reference) | (reference) | (reference) | (reference) |
| Smoking (all) | 0.86 (0.58-1.28) | 0.97 (0.63-1.50) | 0.56 (0.08-4.17) | 1.56 (0.82-2.99) |
| <=10 cig./day | 0.75 (0.44-1.31) | 0.85 (0.47-1.55) | NE | 0.87 (0.28-2.73) |
| 11-20 cig./day | 1.05 (0.55-2.02) | 1.09 (0.52-2.30) | 1.80 (0.24-13.45) | 2.18 (0.81-5.89) |
| >20 cig./day | 0.98 (0.14-6.78) | 1.31 (0.19-9.05) | NE | NE |
|  |  |  |  |  |

^1^Adjusted for sex, age (continuous), education, income (continuous), occupational risk, country of birth and cohabitation

NE= Not estimated because of low numbers of events

# Table S4. Adjusted^1^ risk ratio of COVID-19^2^ for smokers compared with non-users of tobacco, by time period^3^ during the pandemic (N_01/02-31/05_ = 365 600, N_01/06-30/10_ = 365 496)

| Tobacco use | All diagnoses of Covid-19  N= 5 595 | **February 1 to May 31, 2020**  N= 2 135 | **June 1 to October 22, 2020**  N= 3 460 |
| --- | --- | --- | --- |
|  | RR (CI) | RR (CI) | RR (CI) |
|  |  |  |  |
| No tobacco use | (reference) | (reference) | (reference) |
| Smoking (all) | 0.68 (0.61-0.75) | 0.59 (0.49-0.70) | 0.75 (0.66-0.86) |
| <=10 cig./day | 0.80 (0.71-0.90) | 0.61 (0.49-0.76) | 0.93 (0.80-1.08) |
| 11-20 cig./day | 0.44 (0.35-0.56) | 0.49 (0.35-0.68) | 0.40 (0.29-0.58) |
| >20 cig./day | 0.28 (0.13-0.63) | 0.30 (0.10-0.93) | 0.29 (0.09-0.89) |

| Tobacco use | All hospital admissions  N= 1 244 | **February 1 to May 31, 2020**  N= 997 | **June 1 to October 22, 2020**  N= 247 |
| --- | --- | --- | --- |
|  | RR (CI) | RR (CI) | RR (CI) |
|  |  |  |  |
| No tobacco use | (reference) | (reference) | (reference) |
| Smoking (all) | 0.60 (0.47-0.76) | 0.62 (0.48-0.81) | 0.50 (0.28-0.90) |
| <=10 cig./day | 0.63 (0.46-0.85) | 0.62 (0.44-0.87) | 0.65 (0.33-1.27) |
| 11-20 cig./day | 0.52 (0.33-0.80) | 0.51 (0.31-0.84) | 0.53 (0.20-1.42) |
| >20 cig./day | 0.29 (0.07-1.16) | 0.35 (0.09-1.42) | NE |

^1^Adjusted for sex, age (continuous), education, income (continuous), occupational risk, country of birth and cohabitation

^2^ ICU admissions and deaths were too few to be analysed separately

^3^ Until June 1, 2020 testing for SARS-COV-2 was only performed upon medical prescription. From June 1 onwards testing was available on personal request.

NE= Not estimated because of low numbers of events

# Table S5. Adjusted^1^ risk ratio of COVID-19 for smokers compared with non-users of tobacco, by occupational risk of infection (N_low and middle_ = 291 479, N_high_ = 49 878)

| **Occupational risk**  Tobacco use | All diagnoses of Covid-19 | Hospital admission | ICU admission | Death |
| --- | --- | --- | --- | --- |
|  | RR (CI) | RR (CI) | RR (CI) | RR (CI) |
| **Low and middle risk** | N= 3 763 | N= 1 023 | N= 119 | N= 182 |
| No tobacco use | (reference) | (reference) | (reference) | (reference) |
| Smoking (all) | 0.66 (0.58-0.76) | 0.66 (0.50-0.85) | 0.46 (0.20-1.04) | 0.94 (0.51-1.74) |
| <=10 cig./day | 0.80 (0.68-0.94) | 0.68 (0.48-0.95) | 0.56 (0.21-1.54) | 0.64 (0.24-1.73) |
| 11-20 cig./day | 0.41 (0.30-0.56) | 0.54 (0.33-0.89) | 0.22 (0.03-1.61) | 1.06 (0.39-2.88) |
| >20 cig./day | 0.26 (0.10-0.70) | 0.37 (0.09-1.48) | NE | NE |
| **High risk** | N= 1 509 | N= 166 | N= 26 | N= 6 |
| No tobacco use | (reference) | (reference) | (reference) | (reference) |
| Smoking (all) | 0.73 (0.60-0.87) | 0.47 (0.25-0.90) | 0.65 (0.15-2.77) | 3.61 (0.63-20.82) |
| <=10 cig./day | 0.80 (0.65-1.00) | 0.46 (0.20-1.05) | 1.04 (0.24-4.44) | 3.36 (0.36-31.13) |
| 11-20 cig./day | 0.56 (0.38-0.82) | 0.59 (0.22-1.59) | NE | 4.71 (0.50-43.88) |
| >20 cig./day | 0.23 (0.03-1.64) | NE | NE | NE |

^1^Adjusted for sex, age (continuous), education, income (continuous), country of birth and cohabitation

NE= Not estimated because of low numbers of events

# Table S6. Adjusted^1^ risk ratio of COVID-19 for smokers compared with non-users of tobacco among individuals with or without selected diagnoses^2^ of chronic diseases causally associated to tobacco use (N_no diagnosis_ = 264 005, N_diagnosis_ = 101 622)

| **Diagnosis of chronic- diseases^2^** | All diagnoses of Covid-19 | Hospital admission | ICU admission | Death |
| --- | --- | --- | --- | --- |
|  | RR (CI) | RR (CI) | RR (CI) | RR (CI) |
| **No diagnoses** | N= 3 837 | N= 401 | N= 37 | N= 11 |
| No tobacco use | (reference) | (reference) | (reference) | (reference) |
| Smoking (all) | 0.70 (0.62-0.80) | 0.33 (0.20-0.56) | 0.59 (0.18-1.95) | NE |
| <=10 cig./day | 0.85 (0.73-0.97) | 0.39 (0.21-0.71) | 1.04 (0.32-3.44) | NE |
| 11-20 cig./day | 0.39 (0.28-0.54) | 0.22 (0.07-0.69) | NE | NE |
| >20 cig./day | 0.37 (0.14-0.99) | NE | NE | NE |
| **At least one diagnosis** | N= 1 758 | N= 843 | N= 121 | N= 178 |
| No tobacco use | (reference) | (reference) | (reference) | (reference) |
| Smoking (all) | 0.63 (0.52-0.76) | 0.73 (0.56-0.96) | 0.35 (0.14-0.86) | 1.11 (0.62-1.96) |
| <=10 cig./day | 0.70 (0.55-0.89) | 0.78 (0.54-1.11) | 0.40 (0.13-1.27) | 0.81 (0.33-1.99) |
| 11-20 cig./day | 0.53 (0.37-0.74) | 0.64 (0.40-1.04) | 0.20 (0.03-1.44) | 1.31 (0.54-3.22) |
| >20 cig./day | 0.19 (0.05-0.76) | 0.39 (0.10-1.58) | NE | NE |

^1^Adjusted for sex, age (continuous), education, income (continuous), occupational risk, country of birth and cohabitation

^2^ Cancer (all sites), Cardiovascular diseases, COPD, Diabetes mellitus type 2, Parkinson disease

NE= Not estimated because of low numbers of events

# Table S7. Adjusted^1^ Risk Ratio (RR) and 95% Confidence Intervals (CI) of COVID-19 for current smokers, users of snus and mixed users compared to non-users of tobacco among clients of the public dental care clinics in Stockholm region (N = 411 111)

|  |  | **Diagnoses of Covid-19** | **Hospital admission** | **Intensive care** | **Death** |
| --- | --- | --- | --- | --- | --- |
|  |  | RR  (95% CI) | RR  (95% CI) | RR  (95% CI) | RR  (95% CI) |
| **Current tobacco Use**  No use  Exclusive smoking  Exclusive *Snus* Use  Mixed use (smoking and *snus* use) |  | (ref)  0.68 (0.61-0.75)  1.10 (1.01-1.19)  0.67 (0.51-0.88) | (ref)  0.59 (0.47-0.75)  0.95 (0.77-1.17)  0.55 (0.28-1.11) | (ref)  0.43 (0.21-0.88)  0.89 (0.52-1.52)  1.10 (0.35-3.47) | (ref)  0.98 (0.56-1.74)  0.59 (0.26-1.35)  1.57 (0.39-6.40) |

^1^Adjusted for sex, age (continuous), education, income (continuous), occupational risk, country of birth and cohabitation

# Figure S1. Directed Acyclic Graph (DAG) of the hypothesized causal link between tobacco use and COVID-19


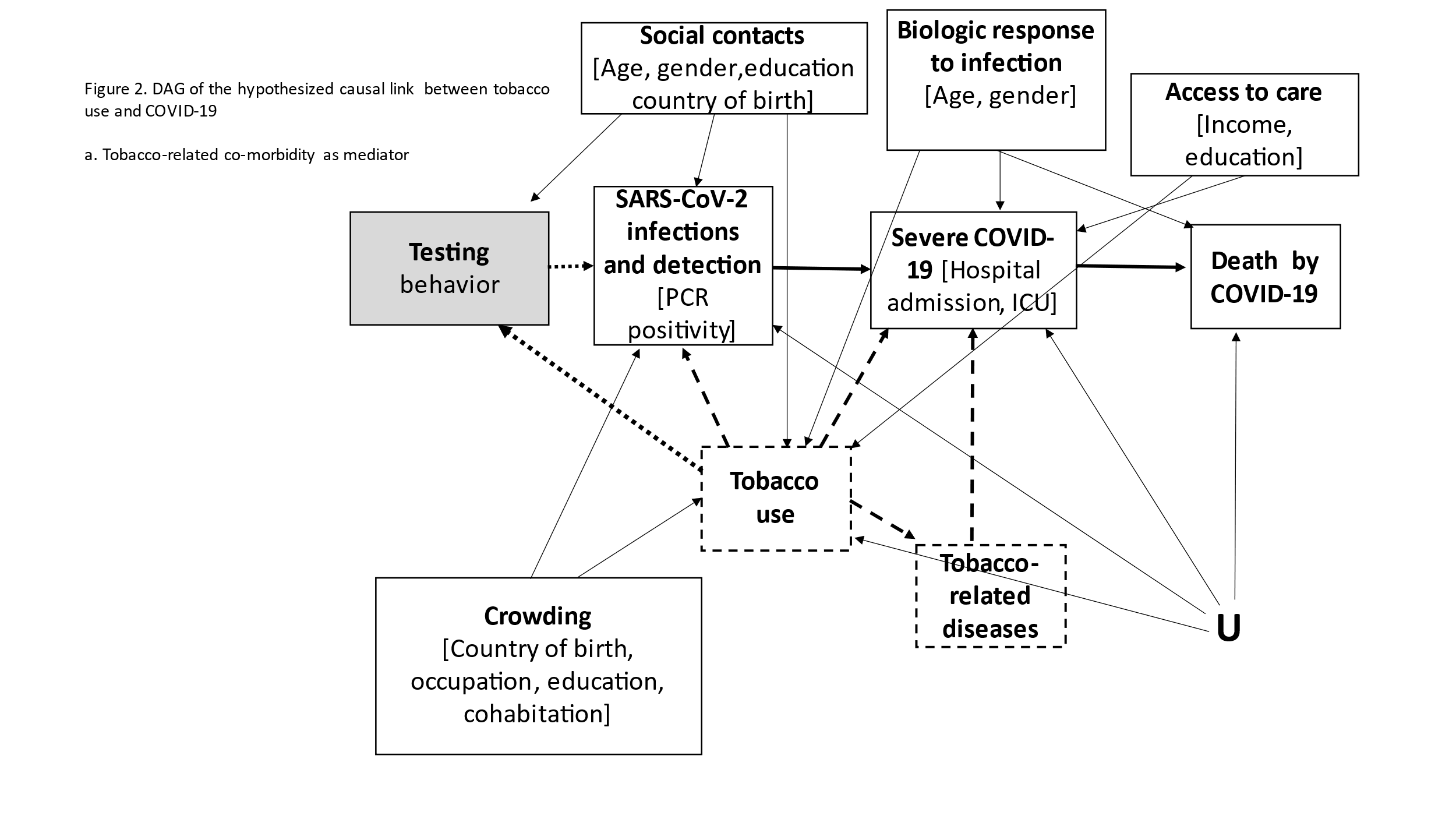


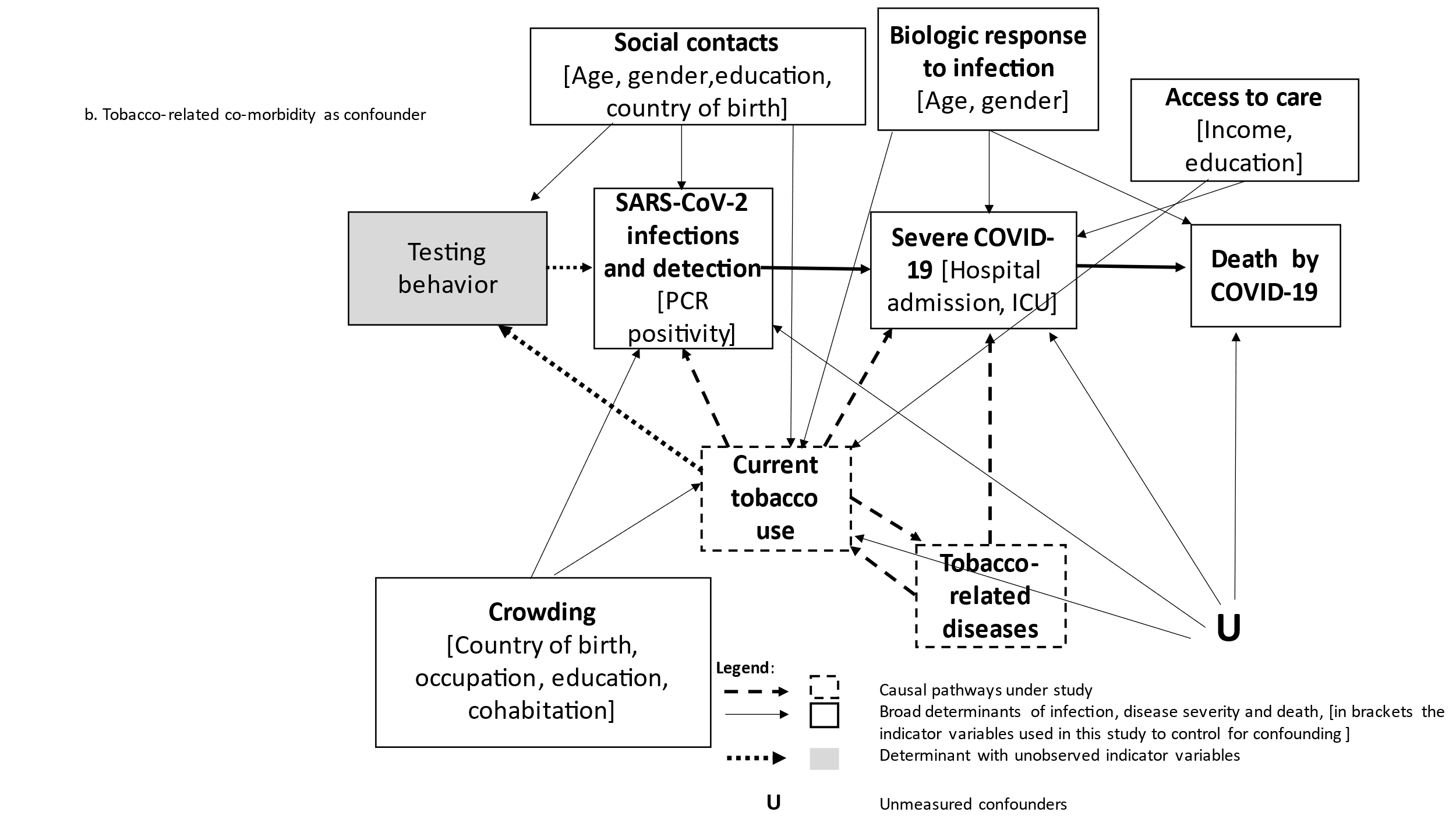

Supplement: Supplementary file 1 — Supplementary Information. [file 41598_2023_28091_MOESM1_ESM.docx]
